# Uncorrected refractive errors for distance among the residents in 'homes for the aged' in South India–The Hyderabad Ocular Morbidity in Elderly Study (HOMES)

**DOI:** 10.1111/opo.12684

**Published:** 2020-03-23

**Authors:** Srinivas Marmamula, Navya Rekha Barrenkala, Rajesh Challa, Thirupathi Reddy Kumbam, Satya Brahmanandam Modepalli, Ratnakar Yellapragada, Madhuri Bhakki, Rohit C Khanna, David S Friedman

**Affiliations:** 1 Allen Foster Community Eye Health Research Centre, Gullapalli Pratibha Rao International Centre for Advancement of Rural Eye care, L V Prasad Eye Institute, Hyderabad, India; 2 Brien Holden Institute of Optometry and Vision Science, L V Prasad Eye Institute, Hyderabad, India; 3 Wellcome Trust/Department of Biotechnology India Alliance, L V Prasad Eye Institute, Hyderabad, India; 4 School of Optometry and Vision Science, University of New South Wales, Sydney, Australia; 5 Department of Ophthalmology, Massachusetts Eye and Ear, Harvard Medical School, Boston, USA

**Keywords:** refractive errors, elderly, residential care, India, spectacle use

## Abstract

**Purpose:**

To investigate the prevalence and risk factors of Uncorrected Refractive Errors (URE) for distance in elderly residents in ‘homes for the aged’ in Hyderabad, India.

**Methods:**

Individuals aged ≥60 years and residing in ‘homes for the aged’ in Hyderabad, India for a minimum of 1 month and providing consent for participation were recruited. All participants underwent visual acuity assessment, refraction, slit lamp biomicroscopy, intraocular pressure measurement, fundus examination, and retinal imaging. Monocular presenting visual acuity was recorded using a logMAR chart. Objective and subjective refraction were performed, and best-corrected visual acuity was recorded. URE was defined as presenting visual acuity worse than 6/12 but improving to 6/12 or better with refraction. Univariable and multivariable logistic regression analyses were used to assess the risk factors associated with URE.

**Results:**

In total, 1 513 elderly participants were enumerated from 41 homes of which 1 182 participants (78.1%) were examined. The mean age of participants was 75.0 years (standard deviation 8.8 years; range: 60–108 years). 35.4% of those examined were men and 20.3% had no formal education. The prevalence of URE was 13.5% (95% CI: 11.5–15.5; *n* = 159). On applying multiple logistic regression analysis, compared to those living in private homes, the odds of URE were significantly higher among the elderly living in the aided homes (OR: 1.65; 95% CI: 1.11–2.43) and free homes (OR: 1.67; 95% CI: 1.00–2.80). As compared to those who reported having an eye examination in the last 3 years, the odds of URE were higher among those who never had an eye examination in the last three years (OR: 1.51; 95% CI: 1.07–2.14). Similarly, those who had unilateral cataract surgery (OR: 1.80; 95% CI: 1.10–2.93) or bilateral cataract surgery (1.69; 95% CI: 1.10–2.56) had higher odds of URE compared to those elderly who were not operated for cataract. Gender, self-report of diabetes, and education were not associated with URE.

**Conclusions:**

A large burden of URE was found among the residents in the ‘homes for the aged’ in Hyderabad, India which could be addressed with a pair of glasses. Over 40% of the residents never had an eye examination in the last three years, which indicates poor utilisation of eye care services by the elderly. Regular eye examinations and provision of spectacles are needed to address needless URE for distance among the elderly in residential care in India.

## Introduction

Visual impairment affects over 253 million people worldwide. Approximately, 80% of the visually impaired people are aged 50 years or older.^1^ Uncorrected Refractive Error (URE) is responsible for nearly half of all visual impairment worldwide, affecting over 124 million people.^2^ Most studies on URE in India were conducted on younger adults where a high prevalence of URE is reported.^3^ A systemic review published recently reported a prevalence of URE as 10.2% among those aged 30 years and older in India.^3^ Data on URE in elderly populations (aged 60 years and older) in residential care in India are limited. We have reported a large burden of URE (15%) among the elderly in residential care in a rural district in India.^4^

India is experiencing a demographic transition resulting in an increase in the proportion of elderly in the population.^5^ One out of every five people is estimated to 60 years or older in India by the year 2050.^5^ There has also been a shift from the traditional joint family systems, in which two to three generations of people lived together, to a nuclear family system with single families, resulting in a rapid rise in the number of homes for aged people in India.^6,7^ The elderly living in these homes are a vulnerable population and previous studies in India and other countries have reported a high prevalence of visual impairment in this group.^4,8-11^.

In urban India, factors such as the complexity of ageing and associated infirmity, ageing singly, and having to navigate unfamiliar urban spaces and procedures are increasingly leading to dependence on others, often necessitating a shift to the 'homes for the aged'. The 'homes for the aged' are a recent phenomenon in India and hence not a well-organised sector. The homes are diverse both in terms of scope, amenities provided and the number of elderly living in them. These homes are typically run by non-government, religious or voluntary organisations with support from the government and philanthropists (free and subsidised homes). In private homes, either the elderly person or their kin pay the ‘user fee’. Most of these homes offer food and accommodation, and private homes have the nursing staff to attend to the medical needs and have other support staff to assist elderly residents in daily routine tasks. There are no eligibility criteria for entry into these homes.

The Hyderabad Ocular Morbidity in Elderly Study (HOMES) aims to provide vital data on visual impairment and other eye conditions in the elderly in residential care in India.^12^ We earlier reported on the burden of visual impairment in this population. 13 The purpose of this paper is to report on the prevalence and risk factors for URE for distance among elderly individuals living in residential care in Hyderabad in South India.

## Methods

### Study setting and study participants

The HOMES study design and procedures were approved by the Institutional Review Board of the Hyderabad Eye Research Foundation, India. The study was conducted in adherence to the Declaration of Helsinki. The HOMES study protocol and sample size estimation have been previously published.^12^ Based on an anticipated prevalence of visual impairment of 15%, a precision of 20% prevalence, a non-response rate of 25%, and a design effect of 1.4 to account for clustering, a sample size of 916 individuals was required. Using the same parameters for sample size calculation and with an anticipated prevalence of URE of 12%, the sample size required for estimation of the prevalence of URE was 1 310 participants.

HOMES was carried out in the home for the aged in Hyderabad and adjoining regions of the Greater Hyderabad Municipal Corporation (GHMC) in the southern Indian state of Telangana. A total of 76 homes were identified within a 50-kilometer radius of L V Prasad Eye Institute (referral centre) of which 46 homes were enrolled for the study. After excluding five homes where the pilot study was conducted, 41 homes were included in the main study. All the residents aged 60 years and older and residing in the homes for at least a period of one month and who agreed to participate were included in the study. Some homes had individuals aged 55 years and older. However, we have not included these younger participants in our study, even though they were examined and were provided with services similar to those aged 60 years and older.

### Eye examination

Detailed personal and demographic information was collected prior to the eye examination. It included age, gender, education, and marital status. The ocular history, including utilisation of eye care services and history of cataract surgery, were recorded. A questionnaire was used to collect information on past and current spectacles use.^13,14^ Self-report of diabetes and hypertension were also collected. The HOMES examination protocol is described in detail in our previous publication.^12^ In short, the eye examination included monocular visual acuity (VA) assessment for distance and near, refraction, slit lamp biomicroscopy, intraocular pressure measurement, undilated fundus examination, and retinal imaging. Monocular presenting VA was recorded in all individuals using a logMAR (Logarithm of the Minimum Angle of Resolution) chart kept at a distance of 3 m under ambient lighting conditions using the letter by letter scoring method. Both English letter optotypes and tumbling E optotype VA charts were used. The VA was tested with the participant's current refractive correction, if used. All subjects underwent objective refraction (manual and autorefraction) and subjective refraction was also performed, and best-corrected visual acuity was recorded.

### Definitions

Visual impairment was defined as presenting distance VA worse than 0.3 logMAR (6/12 Snellen equivalent) in the better eye. This was further subdivided into mild visual impairment (logMAR 0.32 to 0.48 (Snellen equivalent worse than 6/12 to 6/18)); moderate visual impairment [logMAR 0.5–1.0 (Snellen equivalent worse than 6/18 to 6/60)]; severe visual impairment [logMAR 1.02–1.3 (Snellen equivalent worse than 6/60 to 3/60)]; and blindness [logMAR 1.32 to no perception of light (Snellen equivalent worse than 3/60)].^1^ URE was defined as presenting distance VA worse than 6/12 (logMAR 0.3) but improving to 6/12 or better with refraction.

### Data analysis

Data were entered into a database created in Microsoft Access. Data analysis was conducted using Stata Statistical Software for Windows, version 14 (www.stata.com/).^15^ The prevalence of URE was calculated and presented with 95% confidence intervals. Univariable and multivariable logistic regression analyses were used to assess the risk factors associated with URE. Hosmer-Lemeshow goodness of fit test was used to assess the goodness of the model fit. Variance Inflation Factors were used to test for collinearity between the covariates after fitting a multiple regression model. The odds ratio with 95% confidence intervals was calculated. A two-tailed *p*-value <0.05 was considered statistically significant.

## Results

### Study sample

In total, 1 513 elderly participants were enumerated and 1 182 (78.1%) were examined from 41 'homes for the aged' in Hyderabad, India. The mean age of participants was 75 years (S.D.:8.8 years; range: 60–108 years); 64.6% (*n* = 764) of them were women and 20.3% (*n* = 240) had no formal education (*n* = 942). Among those examined, 42.4% (*n* = 510) were from private homes, 41.5% (*n* = 491) from aided/partially paid homes and 16.1% (*n* = 190) from free homes. Only 58% (*n* = 686) reported having undergone an eye examination in the preceding 3 years; 43.3% (*n* = 512) reported having had bilateral cataract surgery and an additional 16.2% (*n* = 191) reported having undergone cataract surgery in one eye (*Table* *1*).

**Table 1:** Personal and demographic characteristics of the participants and Uncorrected Refractive Error (URE)

	Total in the sample *n* (%)^†^	Uncorrected refractive error *n* (%)^‡^
Age group (Years)		
60-69	329 (27.8)	52 (15.8)
70-79	453 (38.3)	63 (13.9)
80 and above	400 (33.8)	44 (11)
Gender		
Male	418 (35.4)	60 (14.4)
Female	764 (64.6)	99 (13)
Education level		
No schooling	240 (20.3)	32 (13.3)
Any education	942 (79.7)	127 (13.5)
Type of home		
Private home	501 (42.4)	50 (10)
Aided/Partially paid	491 (41.5)	78 (15.9)
Free	190 (16.1)	31 (16.3)
Diabetes		
Yes	331 (28)	39 (11.8)
No	851 (72)	120 (14.1)
Duration since last eye exam (years)		
≤3 years	686 (58)	82 (12)
>3 years	496 (42)	77 (15.5)
Cataract surgery status		
No surgery	479 (40.5)	55 (11.5)
Unilateral surgery	191 (16.2)	32 (16.8)
Bilateral surgery	512 (43.3)	72 (14.1)
Total	1182 (100)	159 (13.5)

^†^ Column percentages presented.

^‡^ Row percentages presented.

### Prevalence of uncorrected refractive errors

**Figure 1:**
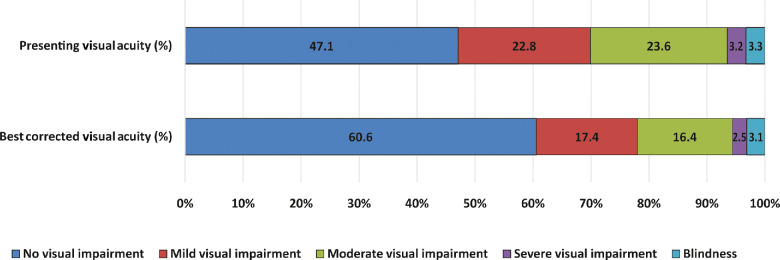
Percentage component bar chart showing presenting and best corrected visual acuity in the better eye (*n* = 1 182).

The prevalence of URE was 13.5% (95% CI: 11.5–15.5; *n* = 159). In total, 104/159 (65.4%) of participants with URE reported having had cataract surgery in one or both eyes; 72 had undergone bilateral cataract surgery. At the time of examination, 92/159 (57.9%) participants who were using spectacles for distance had an inadequate correction of their refractive error. Similarly, 41/159 (25.8%) participants reported using spectacles in the past but stopped using them for reasons such as ‘broken/lost spectacles and cannot afford a new pair (34.1%)’, ‘uncomfortable with spectacles (24.5%)’ and other reasons. As compared to the presenting VA, 295 (25%; 95% CI: 22.5–27.5) participants improved by at least six letters or more with best-corrected visual acuity. Of this, 155 (52.5%) improved by more than one line, 91 (30.8%) improved by more than two lines and 49 (16.6%) improved by more than three lines. Ninety-six participants (8.1%; 95% CI: 6.6–9.8) had presenting VA worse than 6/18 and improved to 6/18 or better with refraction. *Figure* *1* illustrates the visual impairment categories based on presenting and best-corrected VA.

### Risk factors for uncorrected refractive error

On multiple regression analysis, the odds of URE were lower among participants aged 80 and older compared to the younger participants (OR: 0.52; 95% CI: 0.32–0.86). Compared to those living in private homes, the URE was significantly higher among the elderly living in aided homes (OR: 1.65; 95% CI: 1.11–2.43) and free homes (OR: 1.67; 95% CI: 1.00–2.80). Similarly, the odds of URE were higher among those who had not undergone an eye examination in the last three years (OR: 1.51; 95% CI: 1.07–2.14). The participants who had undergone unilateral cataract surgery (OR: 1.80; 95% CI: 1.10–2.93) or bilateral cataract surgery (1.67; 95% CI: 1.10–2.56) had higher odds of URE compared to those who were not operated. Gender, self-report of diabetes and education level were not associated with URE (*p* > 0.05; *Table* *2*).

**Table 2:** Association of uncorrected refractive errors (URE) with socio-demographic characteristics and systemic conditions (Multiple logistic regression analysis) (*n* = 1 182)

	Odds ratio (95% CI) for URE^†,‡,§^	*p*-value
Age group (Years)		
60-69	Reference	
70-79	0.75 (0.49–1.14)	0.18
80 and above	0.52 (0.32–0.86)	0.01
Gender		
Male	Reference	
Female	0.77 (0.54–1.10)	0.16
Education level		
No schooling	Reference	
Any education	1.15 (0.74–1.80)	0.52
Type of home		
Private home	Reference	
Aided/ Partially paid	1.65 (1.11–2.43)	0.01
Free	1.67 (1.00–2.82)	0.05
Diabetes		
No	Reference	
Yes	0.87 (0.55–1.21)	0.32
Duration since last eye exam		
≤3 years	Reference	
>3 years	1.51 (1.07–2.14)	0.02
Cataract surgery status		
No surgery	Reference	
Unilateral surgery	1.80 (1.10–2.93)	0.02
Bilateral surgery	1.69 (11.10–2.56)	0.01

^†^ Based on multiple logistic regression with URE as the outcome variable and all the predictors entered at the same time.

^‡^ Hosmer-Lemeshow test for goodness of fit for the regression model, *p* = 0.144.

^§^ Variance inflation factor for the multiple logistic regression model = 1.10.

## Discussion

Fourteen out of every 100 individuals living in 'homes for the aged' in Hyderabad had URE, highlighting the need for services for the correction of their refractive errors. More importantly, over two-thirds of those with URE had previously undergone cataract surgery, indicating not only suboptimal visual outcomes, but also lack of ongoing follow up care for improved refractive outcomes. Over one-third of these elderly participants never had an eye examination in the preceding three years, it is a worrying finding considering that eye diseases are common in this older population. Our findings highlight a large burden of URE in this vulnerable elderly population in residential care compared to <5% among those of similar age and living in communities in the neighbouring state of Andhra Pradesh, India.^14^

One out of every four elderly individuals living in a home for the aged in our study improved their vision by more than one line (more than five letters) with refraction suggesting the huge unmet need for refraction services in this vulnerable population. A large proportion of those with URE were using spectacles suggestive of inadequate correction due to either poor uptake of eye care services or lack of follow up care. It is also important to note that about one-fourth of those who could benefit from spectacles actually discontinued suggesting the need for more frequent replacement of spectacles. Regular eye examinations for older people are critical not only for the correction of URE but also help in the timely detection of the vision-threatening eye diseases that are common in older people. Currently, there is no policy on eye care for the elderly living in residential homes in India.

Several studies have reported on URE in the elderly including a study that we conducted in the home for the aged in Prakasam district in India.^4,11,16-20^ From a nursing home-based study in the United States, Tielsch reported that 54% of the participants improved their presenting visual acuity on best correction with about 8% improving by three or more lines on the letter chart.^11^ In our study, about 25% of the elderly improved in their presenting visual acuity with the best correction and of which 16.6% had improved by more than three lines on the logMAR chart. However, our study included people aged 60 years and older compared to 40-year-old participants in the US study, hence results are not directly comparable. Another study conducted among nursing home residents in the United States did not find URE as a major cause of visual impairment. This finding may be attributable to the older age of the participants in the US study compared to this study.^21^ Similarly, a recent report among older community-dwelling French individuals found that among those who presented with vision worse than 6/12, 53.7% reported an improvement to 6/12 or better with the best correction which is suggestive of a large a burden of URE.^20^

Several population-based rapid assessment studies conducted in India reported on refractive errors as a major cause of visual impairment. These population-based studies typically included participants aged 40 years or 50 years and older. The nation-wide rapid assessment study in India reported URE as the leading cause of visual impairment among those aged 50 years and older.^22^ In the studies of those aged 40 years and older of the community-dwelling population in the states of Andhra Pradesh and Telangana, URE was the second most important cause of visual impairment.^23,24^

Most of these studies reported from the general population and cannot be extrapolated to those elderly individuals living in residential care settings in India. Another limitation of those studies including the rapid assessment studies is the use of pinhole-based visual acuity improvement as a surrogate measure for URE.^25-27^ Though the pinhole is found to be sensitive to detect URE,^28^ it is subject to certain limitations.^29^ Another challenge in comparing the results across studies is the criteria used to define URE. Some authors used improvement in presenting VA to 6/12 or better while others used 6/18 or better especially in rapid assessment studies. Improvement presenting visual acuity in terms of number of lines is also reported. We reported on improvement of more than one line on a logMAR chart (six letters or more) as this is greater than the test-retest variability reported by Lovie-Kitchin *et al*.^30^ and also provides information on the potential benefit of refractive correction.

We found the odds of URE were lower among the older age groups compared to our earlier study.^4^ This can be explained by a few factors. First, it could be survival bias as only those who are healthy tend to survive longer and studies have shown the association between visual impairment and mortality.^31^ Second, the cause of visual impairment in oldest-old (80 years and older) could be due to other non-correctable causes, due to which they fail to get the best-corrected visual acuity of 6/12 or better. A large proportion of those who had bilateral cataract surgery had URE suggesting the need for follow-up care after cataract for the correction of refractive error. There are not many studies that reported on the burden of URE after cataract surgery in the elderly in residential care in India though the studies have reported on visual outcomes after cataract surgery in the community-dwelling populations.^32-34^ URE was reported as the leading cause of visual impairment after cataract surgery in these studies ranging from 28.7% to 38.8%.

Addressing URE in the elderly requires a different approach compared to cataract programmes. While cataract can be a one-time surgical intervention with intraocular lens implantation, addressing URE needs a regular and repeated intervention to change spectacles as required. Unless URE is adequately corrected and remains to be corrected, the elderly cannot reap the benefits of cataract surgery. A sustainable ongoing programme with an annual eye examination and dispensing of spectacles is recommended. This comprehensive approach will become even more relevant given the demographic shift towards longer life expectancy in India. As the elderly living in 'homes for the aged' form a captive population, screening for vision loss and providing for appropriate intervention are recommended for policy and implementation in practice.

This study had a few limitations. First, we used improvement in visual acuity with the best correction as a measure of URE. However, a proportion of URE can be attributed to index myopia secondary to cataract. Those elderly individuals are likely to benefit from cataract surgery more than spectacles for URE. Second, we reported the burden of URE in the elderly population from the homes for aged and hence the results cannot be extrapolated to the elderly population in the community at large. We also did not record unaided visual acuity which could have helped us to calculate spectacles coverage which is a good outcome indicator for service delivery. Our study included only 41 out of 71 homes in Hyderabad and this could have biased our extrapolations. Near visual impairment is also not reported in this paper. Despite these limitations, due to the strengths of our study such as a large sample size, a good response rate, and a comprehensive assessment, it provides valuable insights on URE status which could help in planning eye care services for the elderly in residential care in India.
